# Neural correlates of language disfunction in psychiatric disorders: a developmental perspective

**DOI:** 10.1007/s00702-026-03232-x

**Published:** 2026-07-30

**Authors:** Mirella Manfredi, Foivos Iliopoulos, Noémie L. Hehl, Susanne Walitza, Nathalie Giroud

**Affiliations:** 1 Department of Child and Adolescent Psychiatry, University Hospital of Psychiatry Zurich, Zurich, Switzerland; 2 Department of Adult Psychiatry and Psychotherapy, University Hospital of Psychiatry Zurich, Zurich, Switzerland; 3 URPP Adaptive Brain Circuits in Development and Learning (AdaBD), University of Zurich, Zurich, Switzerland; 4 Center for Neuroscience Zurich, University and ETH of Zurich, Zurich, Switzerland; 5 Competence Center Language and Medicine, University of Zurich, Zurich, Switzerland; 6 Department of Computational Linguistics, University of Zurich, Zurich, Switzerland; 7 Neurosciences of Communication, Language and Cognition, University Hospital of Psychiatry Zurich, Zurich, Switzerland

**Keywords:** Child psychiatry, Adolesence psychiatry, Language, Social interactions, Speech, EEG, MRI, fNIRS, Development

## Abstract

Language forms the foundation of social interactions, allowing us to connect with family and peers. Through speech, we express our thoughts and emotions to others. Experiencing challenges in speech and language functions during development can therefore significantly affect social relationships, cognitive and affective development. In child and adolescence psychiatry, clinicians have observed that speech and language abilities may be altered in various mental (behavioral/developmental) disorders (psychiatric disorders). This review provides an overview of systematic and original investigations exploring how speech and language are affected in child and adolescent psychiatric conditions, including autism spectrum, schizophrenia, attention deficit/hyperactivity disorder, and depression. We emphasize research that utilized neuroimaging techniques (i.e., EEG, MRI, fNIRS) to investigate the neurobiological basis for alterations in speech or language in child and adolescence psychiatry. The findings indicate that each reviewed syndrome shows evidence of altered speech or language functions, along with corresponding neurobiological changes. Most available studies focus on autism spectrum, while research concerning schizophrenia and depression generally involved adults rather than children or adolescents. This highlights a considerable research gap in understanding (the neurobiological cause of) speech and language alterations in developing populations with psychiatric conditions.

##  Introduction

Language forms the foundation of social interactions and communication and are essential for building social relationships and social connectivity. Sharing thoughts and feelings through various physical signals-such as speech, eye gaze, gesture, and facial expression-enables the transmission of a speaker’s emotional state, ideas, identity or even health status. Communication, most commonly through spoken language, is therefore the primary instrument for human connection and social exchange.

During gestation and childhood, language develops through exposure to specific linguistic environments, while the brain adapts to process this input over time. The intertwined maturation of neural networks and the improvement of language processing with increasing experience underpins a child’s abilities to think, learn, and form parent- and peer relationships (Im-Bolter and Cohen 2007). Thus, the neural basis of language development has been studied extensively over the past two decades due to its central role in neurocognitive and social development (for reviews, see for example Kuhl 2010; Sakai 2005).

In psychiatric clinical practice, alterations in language and communication are frequently observed in children and adolescents with psychiatric conditions, even in the absence of a diagnosed speech or language disorder. To date, little is known about the neurobiological causes or correlates of such changes, or the degree to which alterations in speech or language functions are specific to certain syndromes. Concurrently, rapid technological developments raise growing interest in using the speech signal as a biosignal in precision psychiatry (Kappen et al. 2023). While speech and language parameters have been explored in adults with language disorders (Ruberg et al. 2019) and with psychiatric conditions such as depression (Koops et al. 2023), voice markers have been used to detect conditions like lung or vocal tract cancer (Davis et al. 2022). However, this potential remains largely unexplored in child and adolescent psychiatry.

###  Scope and methodology of this review

The primary goal of this review is to summarize literature exploring changes in speech and language functions and their neurobiological correlates assessed with imaging techniques in children and adolescents with mental disorders. We focus on autism spectrum(AS), schizophrenia, attention deficit/hyperactivity disorder (ADHD), and depression (for the rationale behind this selection and the methodology of this review, see the Review Methodology section below). An overview of the reviewed studies can be found in Table 1. A significant challenge in this endeavor is the scarcity of neuroimaging studies conducted in pediatric populations for some disorders, particularly schizophrenia and depression. This limited evidence likely reflects the relatively low prevalence of childhood-onset forms of these disorders, as well as the fact that clinically relevant symptoms often emerge more clearly during adolescence or early adulthood. In contrast, neurodevelopmental conditions such as autism spectrum and ADHD are typically identified earlier in childhood, facilitating pediatric research. We included studies that (1) collected data of patients performing at least one speech or language task—encompassing both receptive tasks (e.g., speech perception, auditory processing, sentence comprehension) and expressive tasks (e.g., verbal fluency, discourse production, picture naming)— and (2) collected neuroimaging data (MRI, EEG, or fNIRS) from the same individuals, either simultaneously or subsequently. An overview of the reviewed studies can be found in Table 1. We considered studies that deployed magnetic resonance imaging (MRI), electroencephalography (EEG), or functional near-infrared spectroscopy (fNIRS) in addition to the behavioral markers of speech or language functions.

**Table 1:** Overview of reviewed studies

	Authors (Year)	Population	Methods	Core language focus	Main neuroimaging finding
General/Introduction	Im-Bolter and Cohen (2007)	Children	Review	Language impairment & psychiatric comorbidities	Links language development to neurocognitive and social development
	Kuhl (2010)	General	Review	Language acquisition	Describes brain mechanisms in early language acquisition
	Sakai (2005)	General	Review	Language acquisition & brain development	Discusses the interplay between language acquisition and brain maturation
	Kappen et al. (2023)	General	Review	Speech as a biosignal	Proposes speech as a promising biosignal for precision psychiatry
Autism Spectrum Disorder (ASD)	Schaeffer et al. (2023)	ASD	Review/Theoretical	Multilayered language profiles	Proposes three linguistic profiles in ASD: pragmatic deficit, DLD-like, and minimally verbal
	Eigsti et al. (2011)	ASD	Developmental Review	Language acquisition	Frames ASD as a “natural lab” for understanding heterogeneous, non-linear language development
	Gernsbacher et al. (2016)	ASD	Critical Review	“Atypical” language features	Argues that features like pronoun reversal/echolalia are not unique deficits but alternative strategies
	Wang et al. (2025)	ASD children (~ 3 yrs)	hd-EEG, Eye Tracking	Audio-visual speech processing	Found reduced auditory encoding and disrupted audio-visual temporal alignment in ASD
	Larson et al. (2025)	ASD	Systematic Review (fMRI)	Language network connectivity	Pattern of **local hyperconnectivity** in core language regions and **global hypoconnectivity** with social networks
	Groen et al. (2008)	ASD	Integrative Review	Language phenotype & neurology	Links semantic/pragmatic deficits to reduced left lateralization, weak frontotemporal sync, and genetic overlap with SLI
	Haesen et al. (2011)	ASD	Review (ERP/MEG)	Auditory/speech perception	Enhanced low-level processing but impaired integration; atypical right-hemisphere lateralization
	Braeutigam et al. (2008); Fishman et al. (2011)	ASD	MEG/ERP	Semantic processing (N400)	Attenuated/atypical N400 response to semantic incongruities
	Dunn et al. (1999); Manfredi et al. (2020, 2021); Coderre et al. (2018)	ASD children/adolescents	ERP	Semantic processing development	Reduced/delayed N400 effect across modalities, indicating slower maturation of semantic networks
	Sterponi et al. (2015)	ASD	Theoretical	Language as social action	Reconceptualizes “atypical” speech as functional strategies for affective regulation and participation
Schizophrenia (SZ)	Bleuler (1951)	SZ	Seminal Theory	Thought & language	Established language as a window into formal thought disorder
	Covington et al. (2005)	SZ	Linguistic Analysis	Levels of linguistic disturbance	Describes intact phonology/syntax but profound semantic/pragmatic anomalies leading to incoherence
	Docherty (1996); Rochester and Martin (1979)	SZ & at-risk	Behavioral/Discourse	Pragmatics & communication	Pragmatic deficits may be a vulnerability marker present in at-risk individuals
	Nicolson et al. (2000)	Childhood-onset SZ	Clinical/Genetic	Premorbid language impairment	Links premorbid language deficits to higher familial risk, suggesting early atypical neurodevelopment
	Hirano et al. (2020)	SZ	Review (EEG/MEG)	Neurophysiology of language	Summarizes deficits in P50/N100 (gating), MMN (phonology), N400 (semantics), and oscillatory synchrony
	Rapp and Steinhäuser (2013)	SZ	Meta-Analysis (fMRI)	Sentence comprehension fMRI	**Reduced left-hemisphere dominance**: Left fronto-temporal underactivation with compensatory right/bilateral overactivation
	Spironelli et al. (2023)	SZ with AVH	Resting-state fMRI	Language network dynamics	Atypical right-hemisphere recruitment (Broca’s/insula); slow-frequency fluctuations correlate with symptom severity
	Li et al. (2009)	SZ & at-risk	Multimodal MRI Review	Structural language network	Grey/white matter reductions in left language areas; microstructural tract abnormalities; an endophenotype
	Teixeira et al. (2023)	SZ	Computational Review	Digital biomarkers (Speech/EEG)	Reviews automated extraction of linguistic (prosody, coherence) and EEG (complexity, oscillations) biomarkers
ADHD	Redmond et al. (2024); Sarıye et al. (2023)	ADHD	Behavioral	Psycholinguistic abilities	Document co-occurring speech-language processing deficits impacting function
	Luo et al. (2023)	ADHD children (6–8)	EEG	Neural speech tracking	Intact syllable-level tracking; **impaired word-level tracking** (high-gamma) correlating with symptom severity
	Salmi et al. (2020)	ADHD	fMRI (ISC)	Naturalistic speech with distractors	**Desynchronization** in attention/sensory nets with meaningful distractors; divergent recall networks (DMN vs. DAN)
	Ko et al. (2013)	ADHD	fMRI (n-back)	Phonological working memory	Atypical activation: hyperactivation at low load, failure to upregulate fronto-parietal regions at high load
	Kibby et al. (2009)	ADHD/Dyslexia children	Structural MRI	Receptive language & brain volume	Smaller cerebral volume linked to poorer comprehension, relating structure to function across disorders
	Schecklmann et al. (2008)	ADHD	fNIRS (VFT)	Verbal fluency	Reduced prefrontal oxygenation; inverse correlation between brain activity and performance
	Bian et al. (2025)	ADHD	fNIRS (VFT)	Verbal fluency	Reduced DLPFC activation correlating with inattention scores
	Hong et al. (2025)	ADHD	fNIRS (VFT) + ML	Verbal fluency for classification	High classification accuracy using frontal-temporal HbO features
	Zhang et al. (2024)	GAD/ADHD	fNIRS (VFT)	Verbal fluency in comorbidity	Reduced PFC activation in both GAD and GAD/ADHD vs. HC, but no difference between patient groups
	Husain et al. (2023)	ADHD	fNIRS (VFT)	Verbal fluency	Reduced prefrontal activation as a principal diagnostic marker
Depression	Kramer-Ginsberg et al. (1999)	Geriatric MDD	Structural MRI (T2)	Lexical retrieval/verbal fluency	White matter hyperintensities correlate with poorer language task performance
	Sexton et al. (2012)	Late-Life Depression	Structural MRI (DTI)	Cognitive domains incl. language	Reduced FA in frontal-striatal-limbic tracts & corpus callosum linked to executive/speed/language deficits
	Lv et al. (2016)	Bipolar Depression	Resting-state fMRI	Language network connectivity	**Decreased connectivity** between left IFG, MTG, angular gyrus during depression, correlating with symptoms; normalizes in remission
	Koch et al. (2018)	MDD adults	fMRI (Emotional Prosody)	Perception of emotional prosody	Enhanced bilateral amygdala activation to prosody, potentially a depression-specific compensatory marker
	Spironelli et al. (2020)	MDD adults	EEG (Word Tasks)	Phonological/semantic processing	Loss of left-frontal beta asymmetry during tasks, correlating with positive affect and phonological performance
	Fuseda et al. (2022)	Depressed adults	EEG (Natural Speech)	News comprehension & semantics	Delayed early components to positive news; enhanced N400 to negative news; N400 key for classifying depression
	Xie et al. (2019)	MDD adults	Behavioral (Speech-in-noise)	Speech perception in noise	Specific deficit with competing speech (informational masking), indicating increased linguistic interference
	Patil et al. (2023)	Adolescents	Multimodal Deep Learning	MRI + Speech for detection	Hybrid MRI-speech model achieved high depression detection accuracy (trained on adult data)
	Patil et al. (2024)	Adolescents	Federated Learning	MRI + Speech for detection	FL framework integrating MRI & speech data achieved 98% accuracy (trained on adult data)
	Lee et al. (2024)	MDD adults	MRI + NLP of clinical notes	Language use & suicidality	Combined MRI (DMN, midline structures) & NLP (anxiety/agitation topics) best classified suicidal thoughts
	McNeilly et al. (2024)	Adolescents with MDD	Smartphone Lang. + rs-fMRI	Real-life language use	Depressive symptoms linked to pronoun/negative word use and altered DMN/CEN/SN connectivity
	König et al. (2023)	Late Adolescence-Adulthood	Computational Ling. (Avatar)	Spontaneous speech markers	Speech amount and syntactic complexity predicted depression severity (PHQ-8)

*ADHD* Attention-Deficit/Hyperactivity Disorder, *AVH* Auditory Verbal Hallucinations, *DLPFC* Dorsolateral Prefrontal Cortex, *DTI* Diffusion Tensor Imaging, *FA* Fractional Anisotropy, *FTD* Formal Thought Disorder, *fNIRS* functional Near-Infrared Spectroscopy, *GAD* Generalized Anxiety Disorder, *hd-EEG* (high-density EEG, *IFG* Inferior Frontal Gyrus, *ISC* Intersubject Correlation, *MDD* Major Depressive Disorder, *ML* Machine Learning, *MTG* (Middle Temporal Gyrus, *NLP* Natural Language Processing, *ROI* Region of Interest, *rs-fMRI* resting-state fMRI, *SZ* Schizophrenia, *VFT* Verbal Fluency Task

*C/A* Child/Adolescent sample | *A* Adult sample | *Gen* General/not age-specific

*Study includes adolescents but uses adult data/modeling

Before reviewing the empirical literature, it is essential to clarify how “language” is conceptualized in this review. Language is a multidimensional cognitive system that encompasses both receptive and expressive domains, each involving distinct but overlapping neural networks (Hickok and Poeppel 2007; Friederici 2011). Receptive language refers to the ability to comprehend spoken, written, or signed language, including phonological discrimination, lexical access, semantic integration, and syntactic parsing. This domain is primarily supported by the dorsal and ventral streams of the left hemisphere, involving the superior temporal gyrus, middle temporal gyrus, and inferior frontal gyrus (Hickok and Poeppel 2007). Expressive language encompasses the production of language across multiple levels—from articulation and prosody to word retrieval, sentence formulation, and discourse organization. Expressive language relies on overlapping fronto-temporal networks but also involves motor planning regions such as Broca’s area, the supplementary motor area, and the cerebellum (Indefrey and Levelt 2004; Friederici 2011). Importantly, expressive and receptive language are not dissociable in isolation: they operate in dynamic interaction during naturalistic communication, with comprehension providing the input for production and production shaping subsequent comprehension processes (Pickering and Garrod 2013).

Many psychiatric conditions affect these domains differentially. For instance, some disorders may primarily impact expressive pragmatics while leaving receptive semantic processing relatively intact, whereas others may involve early auditory-perceptual deficits that cascade into higher-level comprehension difficulties. Throughout this review, we therefore attend to both receptive and expressive language functions as they are assessed in the reviewed studies. When studies employ speech perception or auditory processing paradigms, we interpret these as tapping into receptive language processes (e.g., phonological and lexical-semantic comprehension). When studies involve verbal fluency, sentence production, or discourse tasks, we interpret these as engaging expressive language processes (e.g., lexical retrieval, syntactic formulation, pragmatic expression). This distinction allows us to map the specific language profiles associated with each disorder onto their neurobiological correlates and to identify disorder-specific patterns of receptive versus expressive language involvement.

This review was conducted as a narrative/scoping review with the aim of mapping the existing literature on neural correlates of language dysfunctions in child and adolescent psychiatric disorders. Given the heterogeneity of the available evidence—spanning different disorders, neuroimaging modalities, and language paradigms—a scoping review approach was considered most appropriate to provide a comprehensive overview of the field and to identify research gaps (Arksey and O’Malley 2005; Munn et al. 2018). We conducted systematic literature searches in the following electronic databases: PubMed, PsycINFO, and Web of Science. Studies were included if they met the following criteria: (1) included participants with a diagnosis of one of the four target disorders (ASD, schizophrenia, ADHD, or depression); (2) included a child or adolescent sample (mean age ≤ 18 years), or, where pediatric literature was sparse (notably for schizophrenia and depression), included adult samples when directly relevant to understanding the disorder’s language profile; (3) employed at least one behavioral measure of speech or language function (receptive or expressive); (4) employed at least one neuroimaging measure (MRI, fMRI, EEG, MEG, or fNIRS) in the same participants, either during task performance or during resting state with a focus on specified language-relevant networks; (5) were published in peer-reviewed journals in English. We excluded case studies, single-case reports, and studies that focused exclusively on non-linguistic cognitive functions (e.g., general executive function without a language component).

The four disorders included in this review were selected based on three criteria: (1) their relatively high prevalence in child and adolescent psychiatry; (2) clinical observations of speech and language alterations as part of their symptomatology; and (3) the existence of at least some neuroimaging research on language functions. We excluded other disorders (e.g., anxiety disorders other than GAD, obsessive-compulsive disorder, eating disorders) where specified language alterations are not a prominent feature or where neuroimaging studies of language are virtually absent. This selection allows for a focused review while maintaining breadth across diagnostically distinct conditions.

Following this methodological overview, Sect.  2, reviews the literature on autism, which has been studied more extensively than other syndromes in this context. Sections  3, 4, and 5, cover the more limited literature on schizophrenia, ADHD, and depression, respectively. In each chapter, we report findings according to neuroimaging modality (MRI, EEG/MEG, fNIRS) and highlight the specific language domains (receptive vs. expressive) assessed. In the final Sect. (6) we summarize the findings, discuss cross-diagnostic patterns, and outline challenges and future directions for integrating language functions into clinical practice and research in child and adolescent psychiatry.

Table 1 presents an overview of studies included in this narrative/scoping review. Studies are organized by disorder and, within each disorder, by neuroimaging modality and language domain (receptive vs. expressive). The table provides a summary of key methodological and conceptual contributions; the full search strategy, inclusion criteria, and selection procedures are described in Sect.  1.1.

## Language in autism

According to the World Health Organization (WHO 2025 [i]) the global prevalence of autism is approximately 1%, although estimates vary across studies, with some recent reports suggesting higher prevalence rates in pediatric populations. Autism spectrum is characterized by persistent difficulties in social communication and social interaction. Common challenges include pragmatic language difficulties, reduced conversational reciprocity, and difficulties adapting communication to social contexts (DSM-5 2022). These challenges primarily affect expressive language in social contexts, though receptive language—particulaly the comprehension of non-literal or context-dependent meaning—is also frequently impacted (Schaeffer et al. 2023).

These communication characteristics make language a central domain of autism research. Following the transition from DSM-IV to DSM-5, growing evidence has highlighted early language abilities, non-verbal intellectual functioning, motor skills, and adaptive behavior (LIMA) as central dimensions for stratifying autism in clinically meaningful and neurobiologically informative ways (Mandelli et al. 2024). These four core features provide a useful conceptual framework for capturing heterogeneity within the autism spectrum beyond a purely continuous view of the condition. In this context, the present work primarily focuses on the type II profile. This subtype generally features more intact or less-impaired early LIMA characteristics. While individuals still meet diagnostic criteria for autism, their non-verbal cognitive and adaptive functional outcomes are much stronger early on.

Research on language in autism spectrum has undergone a paradigmatic shift, from the concept of a single “autistic language” toward a differentiated view recognizing multiple linguistic profiles and heterogeneous communicative strategies. Schaeffer et al. (2023) conceptualize language as a multilayered system (encompassing phonology, morphology, syntax, semantics, and pragmatics) whose components combine differently across autistic individuals. We include their review as a theoretical framework for understanding language profiles in ASD, complementing the neuroimaging studies reviewed below.

The authors identify three main profiles: individuals with structurally intact language but pragmatic difficulties; individuals with language impairments resembling Developmental Language Disorder (DLD); and minimally verbal individuals, whose receptive capacities are often underestimated. These profiles reflect distinct neurocognitive developmental trajectories, with pragmatics as the most vulnerable domain. The authors advocate for personalized clinical approaches that strengthen existing verbal and non-verbal communicative pathways.

Eigsti et al. (2011) adopt a developmental perspective, framing autism as a “natural laboratory” for understanding language acquisition. They highlight the heterogeneous, non-linear trajectory of autistic language development, marked by discontinuities and regressions, contrasting with the uniform milestones of typical development. As noted by Schaeffer (2023), the most consistent difficulties appear in pragmatics (e.g., turn-taking, irony, contextual adaptation), prosody (rhythm, intonation) and syntax, while phonology is typically preserved. Eigsti and colleagues emphasize the reciprocal interactions among language, theory of mind, executive functions, and sensory processing. Notably, electrophysiological studies show that even minimally verbal individuals can exhibit typical neural responses to semantic anomalies, indicating intact implicit linguistic competence. Thus, language in ASD is not merely an output of cognitive or social ability but a dynamic driver of developmental change. From a critical perspective, Gernsbacher et al. (2016) re-examine several features long regarded as linguistic hallmarks of autism (e.g., pronoun reversal, echolalia). They argue that these are not unique to ASD but represent transient phases in typical development or functional communicative strategies. Pronoun reversal, for instance, represents a transient phase also observed in typical development; echolalia, particularly in its mitigated form, constitutes a functional communicative strategy; and comprehension precedes production in autistic as well as typical populations. The authors contend that such “atypicalities” should be interpreted as alternative forms of language organization rather than deficits. Nevertheless, echolalia and pronoun reversal remain important to investigate in autism, as they may emerge in the context of atypical language development and can represent meaningful communicative or self-regulatory strategies, particularly in individuals with limited functional speech.

Early communication difficulties in ASD may stem from impaired auditory and audio-visual integration. In a recent study, Wang et al. (2025) investigated speech processing anomalies in young autistic children (mean age: 3.09 years) using high-density EEG and eye tracking. The study revealed reduced auditory speech encoding and disrupted audio-visual temporal alignment in children with ASD, particularly when visual speech cues were present. These findings indicate that speech processing anomalies, linked to imbalances in multisensory integration, are evident early in development.

###  MRI-based findings

On a neurobiological level, Larson et al. (2025) systematically reviewed fMRI studies investigating connectivity within and beyond the language network in ASD. Their findings converge on a pattern of local hyperconnectivity among core language regions (e.g., between inferior frontal and superior temporal gyri) and global hypoconnectivity with associative and social networks (e.g., insula and somatosensory regions). This architecture may explain the coexistence of preserved structural language skills and persistent pragmatic difficulties. This connectivity pattern appears to evolve with age progressing from local hyperconnectivity in childhood to reduced global integration in adulthood, suggesting a developmentally atypical maturation of integrative networks.

Earlier, Groen et al. (2008) integrated linguistic, neurophysiological, and genetic evidence to outline a similar model. They reported that semantics and pragmatics are most affected, while phonology remains intact. Structurally, the autistic brain exhibits early overgrowth and reduced left-hemisphere lateralization, along with weaker frontotemporal synchronization. These anomalies align with the Weak Central Coherence hypothesis (Happé and Frith 2006), suggesting that autistic individuals tend to process information in a fragmented and locally focused manner. Genetic overlaps with Specific Language Impairment (SLI) further suggest shared neurodevelopmental origins for communication difficulties.

### Neurophysiological findings

Haesen et al. (2011) explored the perceptual underpinnings of the autistic language profile, focusing on auditory processing and speech perception. Behavioral and neurophysiological data revealed enhanced low-level acoustic sensitivity but difficulties integrating temporal and semantic cues, a pattern affecting receptive language at the level of phonological and semantic processing. Electrophysiological responses (ERP, MEG) demonstrate atypical lateralization with right-hemisphere dominance during auditory processing, consistent with the enhanced perceptual functioning model (Haesen et al. 2011). Thus, the challenge in autism may not lie in perception per se, but in the integration of acoustic information into higher-order linguistic representations.

Research on the N400 component, a neural marker of semantic integration, reveals attenuated or atypical responses in autistic individuals to semantic incongruities (Braeutigam et al. 2008; Fishman et al. 2011), suggesting a less automatic and more deliberate integration of meaning during receptive language processing.

 These differences are often accompanied by enhanced late positive components (LPC) and prolonged gamma-band oscillations, markers of explicit cognitive reanalysis.

In autistic children and adolescents, the N400 effect is often reduced and delayed, indicating slower maturation of semantic networks (Dunn et al. 1999; Manfredi et al. 2020, 2021; Manfredi and Coderre 2023). Notably, this reduction occurs across verbal and visual modalities (Coderre et al. 2018), suggesting a domain-general difficulty with contextual integration. This contrasts with expressive language functions such as verbal fluency, which may be relatively preserved in some autistic individuals (Schaeffer et al. 2023).

Overall, these studies indicate that semantic processing in autism is more effortful and analytical. This is consistent with both the Weak Central Coherence and Enhanced Perceptual Functioning models: semantic knowledge itself is intact at the local level but less efficient when contributing in global integration.

Finally, Sterponi et al. (2015) propose a phenomenological reconceptualization, arguing that forms like echolalia, repetition, and ritualized speech are functional strategies for affective regulation and participation, not mere deficits. In this view, they emphasize studying language in naturalistic contexts.

In addition to task-based paradigms, resting-state EEG studies have provided important insights into the neural basis of language development in autism. Resting-state approaches are particularly valuable in young and minimally verbal children because they require minimal task engagement. Previous studies have reported associations between resting-state oscillatory activity and language abilities, particularly within the alpha and gamma frequency bands. Recent work has shown that both aperiodic and periodic EEG features are associated with expressive and receptive language skills in preschool-aged autistic children, with lower aperiodic offset and higher alpha peak amplitude linked to better language outcomes (Chen et al. 2026). Longitudinal findings further indicate that resting-state EEG trajectories differ across autistic language profiles, including language-unimpaired, language-impaired, and minimally verbal children, suggesting that neural oscillatory activity contributes to the heterogeneity of language development in autism (Latrèche et al. 2026). Together, these findings suggest that intrinsic neural activity may represent a promising biomarker of language outcomes in autistic populations.

In summary, evidence across studies delineates a model of language in autism as a differentially integrated neurocognitive system. In the present body of work, focusing on individuals with Type II autism (i.e., without intellectual disability and with relatively preserved formal language), structural language abilities may be preserved, but challenges emerge with increasing semantic and pragmatic complexity, reflecting a cognitive profile characterized by enhanced local processing but weakened global integration.

##  Language in Schizophrenia

According to the WHO (2025), schizophrenia affects approximately 24 million people worldwide, with a prevalence of 0.45% among adults compared to less than 0.01% in children under the age of 15. Schizophrenia is characterized by a range of symptoms including delusions, hallucinations, disorganized thinking, grossly disorganized or abnormal motor behavior, and negative symptoms. Disorganized thinking is typically inferred from an individual’s speech and may manifest as frequent derailment, tangentiality, or incoherence. Consequently, language and communication disturbances represent a central feature of the disorder and are important targets for clinical and neurobiological investigation.

Language in schizophrenia has been discussed for several decades, while more recent neuroimaging studies are rare. Historically, since Bleuler’s seminal work (1951), language in schizophrenia has been considered a window into thought disorder. Linguistic anomalies are not merely epiphenomena of psychotic symptoms but a core component of the illness, revealing disruptions in the architecture of thought. As the interface between mind, body, and society, language is the medium by which meanings are constructed and shared; its disturbance therefore signals a disruption of the link between thought and shared reality.

Over recent decades, the dialogue among linguistics, cognitive neuroscience, and biological psychiatry has clarified the neurocognitive bases of schizophrenic language disturbance, yielding a coherent yet multidimensional picture spanning phonetic, semantic, pragmatic, and neurophysiological levels. Covington et al. (2005) systematically described linguistic disturbances across levels of analysis. Phonetics and phonology, fundamental to both receptive (speech perception) and expressive (speech production) language, are generally intact, though prosody is often flattened. Morphology and syntax are typically grammatically correct but may be simplified, particularly in chronic cases and in the presence of prominent negative symptoms (Morice and Ingram 1982; Thomas et al. 1990).

The most profound anomalies occur at semantic and pragmatic levels, affecting both the comprehension (receptive) and production (expressive) of meaning in context. Semantically, speech may be logically incoherent, driven by clang associations (sound-based links) rather than meaning. This decoupling of linguistic from conceptual content has been framed as a semiotic disturbance (Chaika and Lambe 1986; Wrobel 1989), whereby words no longer anchor to stable referents but drift within associative flow.

Insights into these pragmatic alterations had already been articulated in earlier clinical and linguistic contributions, many of which remain relevant today.

In this context, failures to adhere to conversational principles (Grice 1975), result in tangential, incoherent discourse that primarily reflects expressive pragmatic deficits.

 Classic studies (Docherty 1996; Rochester and Martin 1979) show that such deficits may be present in genetically at-risk, non-psychotic individuals, suggesting a vulnerability marker.

Overall, schizophrenia can feature a dissociation between linguistic form and communicative function: grammar remains intact, yet language loses its capacity to represent reality and regulate thought. As proposed by (Crow 1997; DeLisi 2001), this vulnerability may stem from the very neurogenetic mechanisms that enabled symbolic language in human evolution, a fragility intrinsic to our species’ cognitive complexity.

It is crucial to note that the empirical evidence above is largely derived from adult studies. Our understanding of how these disturbances emerge developmentally is limited. To the best of our current knowledge, only two studies investigated linguistic processing on children and adolescents with schizophrenia. The first study (Baltaxe and Simmon 1995) examined communication characteristics and specific language deficits in a sample of 47 children and adolescents diagnosed with early-onset schizophrenia. Standardized tests and formal assessment measures were employed to evaluate multiple domains of communication, including pragmatics, receptive and expressive vocabulary, syntax, abstract language, auditory processing, and speech production. The findings revealed particularly pronounced impairments in pragmatics, prosody, auditory processing, and abstract language. In the second study, Nicolson et al. (2000) measured 49 patients with childhood-onset schizophrenia and showed that premorbid language impairments were linked to stronger familiar risk factors implying atypical development of language-related brain regions. However, the onset and progression of language alteration during adolescence -a critical period for both neurodevelopment and psychosis risk- remain poorly investigated and warrant future studies. One key reason for this gap is the rarity of early-onset psychosis in adolescence, with childhood-onset schizophrenia representing an exceptionally rare condition. Nevertheless, greater attention to speech abnormalities in this population is warranted, as they may represent relatively unconfounded markers of schizophrenia prior to chronification, allowing researchers to examine language changes from their earliest emergence, including during the transition from at-risk states to full-blown psychosis.

###  MRI-based findings

Functional neuroimaging evidence converges on a pattern of reduced left-hemisphere dominance for language in schizophrenia. Reviewing 13 fMRI studies, Rapp and Steinhäuser (2013) report underactivation of left fronto-temporal regions, accompanied by compensatory bilateral or right-hemisphere overactivation. These patterns correlate with “formal thought disorder (FTD)” and auditory verbal hallucinations (AVH), respectively, and align with Crow’s (1997, 2000) hemispheric disconnection hypothesis.

Extending this line of research, Spironelli et al. (2023) employed resting-state fMRI to demonstrate that patients with AVH exhibit atypical recruitment of right-hemisphere homologues, specifically in the right inferior frontal (Broca’s area) and anterior insular cortex. This finding suggests a state of right-hemisphere hyperactivity coupled with left-hemisphere hypoactivity. Furthermore, slow-frequency fluctuations within the language network were found to correlate with delusion and hallucination severity, respectively, indicating that low-frequency oscillations may serve as functional biomarkers of psychosis vulnerability.

In a multimodal synthesis, Li et al. (2009) documented concurrent reductions in grey and white matter volume within left-lateralized language areas, alongside microstructural abnormalities in associative tracts (e.g., arcuate and uncinate fasciculi; corpus callosum). Crucially, similar patterns are observed in genetically at-risk individuals, reinforcing the view that language network dysfunction is a neurodevelopmental anomaly that precedes clinical onset and constitutes a core endophenotype of the disorder.

### Neurophysiological findings

Neurophysiologically, schizophrenia is associated with EEG/MEG markers across processing stages (Hirano et al. 2020). Deficits in early components (P50, N100) indicate impaired auditory and corollary discharge, mechanisms implicated in AVH. The Mismatch Negativity (MMN), particularly to phonemic stimuli, is reduced, indicating phonological processing deficits. At the semantic level, the N400 shows diminished amplitude and prolonged latency, signaling difficulties in meaning integration and semantic coherence, correlating with symptoms of FTD. Abnormalities in theta and gamma band oscillations further suggest impaired cortical synchronization during language tasks. This impaired synchronization aligns with NMDA receptor hypofunction, indicating potential neurochemical dysregulation within the coupling mechanisms that underpin complex linguistic operations.

Resting-state EEG studies have also contributed to understanding the neurobiology of language dysfunction in schizophrenia. Increased slow-wave activity, particularly within the delta band, has consistently been reported during resting-state conditions and is generally interpreted as a marker of cortical dysfunction and reduced neural integration (Spironelli et al. 2011). Previous studies have identified increased frontal delta activity, especially over left frontal regions, together with reduced hemispheric asymmetry and altered functional connectivity. Spironelli et al. (2011) further demonstrated that increased left frontal delta activity was associated with reduced language lateralization and with positive symptoms such as delusions and conceptual disorganization. These findings support theories proposing that schizophrenia involves disrupted fronto-temporal integration and atypical hemispheric specialization for language, which may contribute to impaired communicative coherence and thought disorder.

###  New directions: computational linguistics and digital biomarkers

Advances in AI and computational methods are catalyzing the search for objective linguistic biomarkers. Teixeira et al. (2023) reviewed progress in automated language and EEG analysis for early detection. Linguistically, patients show reduced prosodic variability, slowed speech with increased pauses, and lower semantic coherence. Speech-emotion recognition consistently reveals blunted affective modulation, consistent with negative symptoms. EEG features, such as nonlinear complexity metrics and oscillatory patterns, show strong discriminative power in machine-learning models, though challenges regarding generalizability and ethics remain. Nonetheless, significant challenges remain regarding cross-linguistic standardization, sample size, generalizability, and the ethical use of AI in clinical settings.

Taken together, evidence from linguistic, EEG/MEG, fMRI, and computational studies suggests that language disturbances in schizophrenia are associated with altered integration within fronto-temporal and language-related networks. Reduced left-hemisphere lateralization, atypical right-hemisphere recruitment, and impaired neural synchronization may contribute to the loss of semantic and communicative coherence that characterizes the disorder. Future studies, particularly in pediatric populations, are needed to clarify the developmental trajectory of these alterations and evaluate their potential as early biomarkers of psychosis risk.

## Findings during speech or language tasks in ADHD

The global prevalence of Attention-Deficit/Hyperactivity Disorder (ADHD) is estimated at approximately 8% in children and adolescents and 3% in adults, as reported in recent global mental health literature aligned with WHO findings. ADHD is frequently associated with deficits in speech and language processing that impact everyday functioning. These include expressive difficulties such as excessive talking, frequent interruptions, difficulty taking turns in conversation, and tangential or disorganized speech (Bruce et al. 2006; Green et al. 2014). Receptive language difficulties are also common, manifesting as poor listening skills, difficulty following instructions, and problems with auditory processing and selective attention in noisy environments (Chermak et al. 2017; Lanzetta-Valdo et al. 2016). Pragmatic language deficits—including inappropriate topic shifts, reduced sensitivity to conversational cues, and difficulties with narrative coherence—are particularly well-documented (Staikova et al. 2013; Helland et al. 2012). These communication challenges are not merely secondary to attentional deficits but appear to reflect overlapping neurocognitive vulnerabilities (Redmond 2016; Mueller and Tomblin 2012).

ADHD is frequently associated with deficits in speech and language processing that impact everyday functional abilities (Redmond et al. 2024; Sarıye et al. 2023; for reviews, see Mueller and Tomblin 2012; Machado-Nascimento et al. 2016; Redmond 2016). However, the number of neuroimaging studies investigating the neural mechanisms underlying these impairments in individuals with ADHD remains remarkably limited, a finding that emerged clearly from our literature search.

### MRI-based findings

Salmi et al. (2020) used fMRI intersubject correlation (ISC) analysis to investigate how children with ADHD process speech during a complex, multi-talker listening task with competing auditory distractors, a paradigm that taps receptive language processing under ecologically challenging conditions. The research revealed altered brain synchronization in individuals with ADHD, specifically desynchronization in attention networks and sensory cortices. A key finding was that this desynchronization in the posterior parietal cortex occurred selectively in the presence of meaningful background distractors, such as speech or music, but not with white noise. This highlights a specific difficulty in filtering complex auditory information. Furthermore, the neural basis for successful memory recall differed between groups; it relied on the default-mode network in ADHD participants, versus the dorsal attention network in controls. In conclusion, these findings demonstrate that ADHD alters large-scale brain network synchronization during naturalistic speech perception, particularly under conditions requiring the suppression of competing linguistic inputs.

In 2013, Ko et al. conducted an fMRI study investigating phonological working memory, a process involving language-related information that supports both receptive (comprehension) and expressive (production), in adults with ADHD. Although their study did not directly assess speech performance or production, it revealed relevant neural correlates. The findings revealed atypical neural activation patterns compared to neurotypical controls. During low-demand conditions, the ADHD group showed hyperactivation in regions including the bilateral anterior cingulate cortex, left inferior frontal gyrus, hippocampus, and supplementary motor area—regions associated with executive control and verbal working memory. However, when task difficulty increased (from a 2-back to a 3-back condition), the ADHD group demonstrated a paradoxical reduction in activation within the left precuneus, insula, and supplementary motor area. In contrast, controls showed the expected increase in engagement of the fronto-parietal network. These findings indicate that adults with ADHD may recruit compensatory neural resources under low cognitive load but exhibit insufficient upregulation of task-relevant regions when cognitive demands escalate. This pattern suggests a dysfunction in dynamic resource allocation and neural scalability during phonological working memory tasks, reflecting broader deficits in the executive control and adaptive modulation of language-related neural circuits in ADHD.

An older fMRI study by Kibby et al. (2009) investigated structural brain correlates of receptive language functioning in children with developmental disorders, including ADHD and dyslexia. Overall cerebral volume did not differ significantly between the diagnostic groups and typically developing controls. However, children exhibiting poor receptive language skills had bilaterally smaller cerebral volumes than those with typical language comprehension, indicating that structural differences are more closely tied to language functioning than to a specific diagnostic classification. Importantly, the relationship between cerebral volume and receptive language ability was non-linear: reductions in brain volume were associated with poorer comprehension only among children with the smallest total brain volumes. These findings suggest that diminished cerebral volume contributes specifically to receptive language impairments, rather than representing a generalized neuroanatomical feature of ADHD or dyslexia. This highlights the relevance of structural variability for understanding language comprehension difficulties across developmental disorders.

###  fNIRS findings

A review of the neuroimaging literature reveals a disproportionate representation of functional Near-Infrared Spectroscopy (fNIRS) in studies of language and speech production, despite the broader dominance of fMRI and EEG for assessing functional brain activity. The rationale for this lies in the significant methodological challenges posed by speech. The requisite orofacial and head movements generate profound motion artifacts in fMRI data and confound EEG signals with electromyographic (EMG) activity. Consequently, fNIRS has emerged as an optimal compromise, enabling the acquisition of reliable hemodynamic data from key language-related regions with considerable resilience to movement artifacts. This methodological advantage has prompted a considerable expansion of fNIRS-based studies focusing on language and speech processing in ADHD, with the verbal fluency task serving as the predominant experimental paradigm.

A seminal study demonstrating the methodological utility of fNIRS in language research was conducted by Schecklmann et al. (2008), who examined brain activity during verbal fluency tasks (VFT) in adults with ADHD relative to healthy controls, leveraging fNIRS’s low susceptibility to movement artifacts. The findings indicated that both groups exhibited inferior frontal brain activation during phonological and semantic verbal fluency. However, adults with ADHD demonstrated a reduced magnitude of prefrontal oxygenation. Notably, an inverse correlation between brain activity and performance was observed in the ADHD patients, whereby higher brain activity was associated with lower verbal fluency output.

A very recent study by Bian et al. (2025) investigated cortical activation during a VFT in children with ADHD. The results revealed significantly reduced hemodynamic activity in the dorsolateral prefrontal cortex (DLPFC) compared to neurotypical controls. This hypoactivation was quantified using multiple parameters, including mean amplitude, center of gravity, and initial slope. In addition to hypoactivation, widespread negative activation patterns were observed, suggesting divergent neural engagement during language production and executive processing. Critically, the mean activation amplitude in the DLPFC demonstrated a significant negative correlation with clinical inattention scores. These findings underscore the role of DLPFC dysfunction in ADHD during language-executive tasks and posit fNIRS as a promising objective tool for quantifying attention-related neural deficits in pediatric populations.

Concurrently, Hong et al. (2025) employed fNIRS during VFT to examine brain activity in adults with ADHD compared to neurotypical controls. The primary objective was to develop a machine learning classifier based on neuroimaging data. The analysis achieved a high classification accuracy of 89.74%, identifying hemoglobin concentration (HbO) features from a combined frontal-temporal region as the most discriminative biomarker for differentiating the groups. This outcome implicates aberrant neural activation within this language-related network during a demanding speech task in adult ADHD. However, the study does not characterize the specific nature of the activation differences (e.g., hypo- or hyperactivation) or provide a detailed functional interpretation of the distinct patterns observed.

Another recent fNIRS study by Zhang et al. (2024) investigated prefrontal cortex (PFC) hemodynamic activity during a VFT in patients with generalized anxiety disorder (GAD) and comorbid GAD/ADHD. The key finding was a significant reduction in oxygenated hemoglobin (HbO) activation in both patient groups compared to healthy controls, localized to the medial PFC and left ventrolateral PFC. Critically, no significant neurofunctional differences were observed between the comorbid GAD/ADHD group and the GAD-only group during the VFT. This result contrasts with the study’s parallel findings from a GO/NOGO task, which differentiated the groups. This suggests that the neural correlates of verbal fluency, as measured by fNIRS, are similarly impaired in GAD and comorbid GAD/ADHD and may lack the specificity to serve as a diagnostic biomarker for distinguishing between these clinical conditions.

In a similar approach, the study by Husain et al. (2023) also implemented verbal fluency tasks in conjunction with fNIRS to assess prefrontal cortex function in adults with ADHD compared to healthy controls. Results demonstrated significantly reduced prefrontal activation in the ADHD group, indicating attenuated cortical engagement during verbal fluency performance. This diminished hemodynamic response emerged as the principal neuroimaging marker differentiating ADHD participants from controls and demonstrated high diagnostic potential, correctly classifying over 75% of participants in both groups.

###  Neurophysiological findings

In a recent EEG study, Luo et al. (2023) investigated neural speech tracking at the syllable and word levels—a paradigm probing receptive speech processing. Children aged 6–8 listened to rhythmic speech sequences in which syllables and words were presented at distinct frequencies, allowing distinct neural responses to be attributed to each linguistic unit. The analysis revealed reliable neural tracking for both syllables and words in the low-frequency band (< 4 Hz), indicating that basic acoustic and rhythmic receptive speech processing remain intact in this age group. Similarly, robust tracking was observed in the high-gamma band (70–160 Hz), which is, according to the authors, associated with cortical computations for higher-order linguistic processing. The strength of high-gamma tracking was negatively correlated with ADHD symptom severity suggesting weaker neural encoding of linguistic information. Based on the current literature, the aforementioned study appears to be the sole EEG investigation evaluating brain activity during speech-related tasks in individuals with ADHD, whereas a slightly larger body of fMRI research has been conducted in this population. This limited evidence also extends to the resting-state EEG literature. Although resting-state EEG has been widely used to investigate neural abnormalities in ADHD, only a small number of studies have examined these measures in relation to language, reading, or speech-related abilities.

### Summarizing conclusion

Taken together, the existing neuroimaging evidence indicates a dual-level characterization of ADHD-related language processing deficits. Functionally, ADHD is associated with impaired higher-order linguistic integration and filtering, alongside divergent recruitment of prefrontal and attentional networks. This is particularly evident under conditions of increased cognitive load or competing auditory input. Clinically, these neural alterations correspond to observable language and attention difficulties. Prefrontal hypoactivation and atypical network synchronization show promise as potential objective biomarkers, although their diagnostic specificity requires further investigation. Furthermore, structural variability in cerebral volume modulates language comprehension outcomes. This evidence emphasizes that ADHD-related impairments in speech and language processing reflect a combination of dynamic functional disruptions and individual neuroanatomical differences, rather than generalized deficits across all auditory or language domains.

## Depression

Major depressive disorder (MDD) is a common psychiatric diagnosis, with an estimated prevalence of approximately 4% worldwide (WHO 2025 [ii]). Beyond affective and motivational disturbances (Marx et al. 2023), the disorder is associated with marked alterations in cognitive (Pan et al. 2019) and language-related processes (Corbin et al. 2023; Trifu et al. 2017, 2024). Depression is characterized by persistent low mood, anhedonia, and changes in appetite, sleep, and energy (DSM-5 2022). Communication difficulties in depression manifest across both expressive and receptive domains. Expressive language changes include reduced verbal output (alogia), prolonged speech latencies, flattened prosody, decreased speech rate, and increased use of first-person singular pronouns and negative emotion words (Rude et al. 2004; Brockmeyer et al. 2015; Koops et al. 2023). Clinically, these changes may present as impoverished or slowed speech, reduced pitch variability, and monotonous intonation that can be perceived as lacking emotional engagement (Cannizzaro et al. 2004; Mundt et al. 2012). Receptive language difficulties include impaired perception of emotional prosody, reduced comprehension of affective speech cues, and increased susceptibility to linguistic interference, particularly in noisy environments or when processing negative content (Xie et al. 2019; Koch et al. 2018). These communication alterations are thought to reflect both the psychomotor retardation and the cognitive biases characteristic of depressive states (Beck 2008; Trifu et al. 2024).

Over the past decade, an increasing number of studies have examined the neural correlates of language functions in depression using neuroimaging, electrophysiological, and behavioral approaches. It should be noted that although the current review focuses on studies conducted on children and adolescents, our systematic literature search identified virtually no neuroimaging studies of language in pediatric depression. Therefore, in this chapter we review findings from adult and late-life populations to provide an overview of the neural correlates of language in depression, while explicitly highlighting the critical gap in pediatric research as a key finding of this review.

### MRI-based findings

Several neuroimaging studies have investigated how speech and language processes are affected in adult individuals with depressive disorders. They highlight the role of both structural and functional abnormalities in brain networks related to linguistic and cognitive function. Kramer-Ginsberg et al. (1999) examined T_2_-weighted MRI scans in individuals aged 65 years or older diagnosed with geriatric unipolar depression and compared them to a healthy, age-similar sample. They found that greater white matter hyperintensities correlate with poorer performance on expressive language tasks, including lexical retrieval (Boston Naming Test) verbal fluency (Animal Naming Test), and memory recall (general memory and delayed recall index). Their findings provide early evidence that, within an elderly population, structural white matter pathology may contribute to expressive language and cognitive impairments associated with depressive symptoms.

Sexton et al. (2012) similarly investigated the neuroanatomical correlates of neuropsychological deficits in late-life depression (LLD). Their study compared performance across five cognitive domains, namely episodic memory, executive function, language skills, processing speed and visuospatial skills, between LLD patients and healthy controls. Patients with LLD demonstrated significantly poorer performance in executive function, processing speed, episodic memory, and language skills, while visuospatial abilities remained intact. These cognitive deficits were linked to reduced fractional anisotropy (FA) in frontal white matter tracts, particularly within frontal-striatal-limbic connections supporting executive function and the genu of the corpus callosum (linked to processing speed). This association emphasizes the role of disrupted frontal-cortical connectivity in mediating the slowed information processing and linguistic inefficiency characteristic of LLD. Additionally, deficits in executive function and processing speed were identified as core cognitive features of LLD, leading to impairments in other cognitive domains.

Additionally, two functional MRI (fMRI) studies have investigated language-related network dysfunction in both unipolar and bipolar depressive disorders. Lv et al. (2016) employed resting-state fMRI to compare functional connectivity between adult patients with bipolar disorder and healthy controls. Connectivity was assessed across 90 predefined language-related regions of interests (ROIs) during both depressive episodes and states of remission. Their analysis revealed significantly decreased functional connectivity between several key left hemispheric language regions, including the inferior frontal gyrus, middle temporal gyrus and angular gyrus in patients experiencing a depressive episode. Furthermore, diminished activity was observed in other brain regions functionally connected to these language nodes. Decreased connectivity strength of several regions showed a significant correlation with the severity of depressive symptoms. The authors propose that this disrupted connectivity underlies the clinical phenomena of “slowed thinking” and psychomotor retardation in bipolar depression. Notably, connectivity strength in patients during remission did not differ significantly from that of healthy controls.

Koch et al. (2018) investigated the impaired perception of emotional prosody, defined as a receptive language function involving the comprehension of paralinguistic cues, in adult patients with major depressive disorder (MDD) using fMRI. Emotions were conveyed in the form of happy, neutral and sad prosodic stimuli. Both depressed patients and controls exhibited increased activation in the superior and middle temporal gyri, regions central to speech and language processing, and the amygdala when listening to emotional versus neutral prosody. MDD patients showed heightened bilateral amygdala activation than healthy controls, independent of emotional prosody. Furthermore, a generally enhanced activation in the left amygdala was observed in MDD, which correlated with a less pronounced negative attribution bias of happy prosody. The authors interpreted the enhanced amygdala response as a potential depression-specific marker. The response potentially suggests a compensatory emotion-regulation process that facilitates accurate perception of emotional prosody.

### Neurophysiological findings

Two EEG studies have provided complementary insights into how depression alters the neural dynamics of speech and language processing, revealing abnormalities in both controlled linguistic tasks and naturalistic listening contexts.

Spironelli et al. (2020) employed EEG to test whether major depression in adult patients is associated with left frontal hypofrontality during both resting-state conditions as well as phonological and semantic word tasks. Frontal alpha asymmetry did not differ between healthy controls and MDD patients during resting state, with no evidence of clear lateralization. Similarly, resting-state beta activity showed a largely bilateral pattern, although MDD patients displayed reduced beta amplitude over left frontal regions, suggesting diminished prefrontal activation. During task performance, healthy controls exhibited the expected left-lateralized beta activity over frontal regions, whereas MDD patients showed a bilateral activation pattern with reduced left frontal beta amplitude. The more symmetrical pattern was accompanied by poorer performance in phonological processing, while semantic processing remained relatively intact. The authors interpreted this pattern as a subtle yet specific linguistic impairment. Moreover, left frontal beta activation correlated positively with positive affect, linking reduced frontal activation to diminished emotional and cognitive engagement.

In a complementary line of research, Fuseda et al. (2022) investigated EEG responses to naturalistic spoken language of positive, neutral, and negative radio news clips, in adults with elevated depressive symptoms compared to non-depressed controls. Across groups, the amplitude of the P2 component was significantly larger for negative than for positive news, indicating a general negativity bias and elevated attentional engagement with negative content. Depressed participants reported lower arousal and comprehension across conditions. Critically, EEG analysis revealed disorder-specific patterns: depressed individuals exhibited delayed early components (N1, P2) in response to positive stimuli and an enhanced N400 amplitude to negative content. This pattern suggests slower attentional allocation to positive speech and increased semantic processing demands for negative speech in depression. Furthermore, a Support Vector Machine (SVM) classifier trained on EEG features identified the N400 amplitude elicited by negative news as the most discriminative feature for classifying depressive states. This finding underscores the potential relevance of neural correlates of semantic processing during natural speech comprehension as objective markers for identifying depression.

### Behavioral approach

Xie et al. (2019) investigated how MDD affects speech perception in varying background noise conditions that differed in linguistic content (high, low, none). Compared to healthy controls, participants with MDD demonstrated reduced key-word recognition accuracy, but only when target sentences were masked by meaningful competing speech. Accuracy was not significantly impaired when masking involved spectrally rotated speech or speech-shaped noise. A speech recognition error analysis revealed that depressed individuals frequently reported words from the background talker, which the authors interpreted as evidence of increased linguistic interference. Overall, individuals with MDD exhibited a specific deficit understanding speech in the presence of competing talkers (informational masking), indicating increased vulnerability to linguistic distraction.

### New directions: multimodal and computational approaches

Recent studies have integrated neuroimaging with computational models of speech and text to identify multimodal biomarkers of depressive disorders. Patil et al. (2023) developed a multimodal deep learning model that combines MRI brain imaging and speech features to detect depression in adolescents. Each modality was extracted and classified separately via a Deep Maxout Network (DMN). The DMN was trained using a nature-inspired optimization algorithm. The outputs from the MRI- and speech-based classifiers are then fused to generate the final classification as “healthy” or “depressed”. This hybrid approach achieved high performance and outperformed several traditional machine learning and deep learning models.

Building on this, Patil et al. (2024) applied a Federated Learning (FL) framework to overcome large-scale data limitations in integrating MRI and speech data for multimodal depression detection in adolescents. Within the FL framework, data were processed locally on multiple devices, where MRI and speech data were handled separately and model training was again guided by a nature-inspired optimization algorithm. The locally trained models were subsequently aggregated on a central server to generate a final classification. Using MRI and speech data from the MPI-Leipzig-Mind-Brain-Body dataset, the proposed framework reached 98% accuracy. The results demonstrate the potential of a scalable, data-protected framework that integrates MRI and speech data across institutions. However, it should be noted that in both Patil et al. (2023) and Patil et al. (2024), the models were trained and evaluated exclusively on datasets consisting of adult participants, despite being framed as targeting depression detection in adolescents.

Beyond acoustic data, Lee et al. (2024) combined structural MRI data with natural language processing (NLP) of psychiatric intake notes to classify suicidal thoughts at the time of depression diagnosis in adult patients. This integrative approach outperformed text-only and MRI-only models. NLP features that contributed most to the classification included anxiety- and agitation-related conversation topics. Although no significant differences were observed in overall structural network patterns between the suicidal and non-suicidal groups, group differences emerged in mean MRI loading weights within the default mode network (DMN), particularly in the precuneus, and the cortical midline network, especially in the anterior cingulate cortex. The authors noted that these regions are associated with self-referential processing, rumination, and negative self-image.

McNeilly et al. (2024) extended this multimodal approach to adolescents by linking real-life smartphone-based language (passively collected through keyboard data) to resting-state fMRI connectivity. The study examined depression-related language use in adolescents with and without MDD. Depressive symptoms correlated with increased daily use of first-person pronouns and negative emotion words, reduced use of future-focused language, and altered connectivity within and between large-scale networks. Specifically, a greater DMN connectivity correlated with higher use of first-person pronouns and a higher proportion of future-focused words. Additionally, greater left central executive network connectivity was linked to more frequent first-person pronoun use. Finally, stronger salience network connectivity was associated with increased use of negative emotion words.

Ultimately, König et al. (2023) demonstrated that spontaneous speech produced during an interaction with a virtual avatar can predict depression severity, by implementing measures of expressive language production. The dataset underlying their analyses included individuals ranging from late adolescence to older adulthood. Their best regression model explained approximately 10% of the variance in depression severity scores (Patient Health Questionnaire PHQ-8 total score), with produced speech amount and complexity of syntactic structure emerging as key linguistic markers of expressive language output. Moreover, the regression model had an average prediction error of 1.25 points, which means that the model could reasonably distinguish meaningful differences in depression levels between participants.

## Discussion

Our review suggests that both receptive and expressive speech and language functions are altered across all studied diagnostic and age groups (ASD, schizophrenia, ADHD, depression), though comprehensive data is lacking. However, the specific domains affected and the degree to which receptive versus expressive processes are involved vary considerably across disorders and remain incompletely characterized in pediatric populations. The systematic identification and mapping of the existing evidence, which wasthe core aim of our scoping approach, reveals substantial heterogeneity in study designs and a notable scarcity of pediatric neuroimaging studies for some disorders.

The neurobiological mechanisms behind these changes remain unclear, especially in children and adolescents, but initial studies indicate distinct neurobiological underpinnings between syndromes. Nevertheless, the studies identified in our review indicate distinct neurobiological underpinnings between syndromes, though the heterogeneity of methods across studies limits direct comparison. There is a clear need to understand how mental disorders affect speech and communication during development, as such changes may serve as diagnostic markers. They are crucial for predicting learning as well as social and cognitive development and should be considered in behavioral treatment approaches such as in psychotherapy.

In sum, language in ASD, especially in the high functioning ASD group, has been studied more extensively than in other child and adolescence psychiatric conditions. Given the current state of the literature, it is assumed that the auditory and the language system are independently functionally over-synchronized, while weakly integrated with each other and other neural systems leading to various changes in pragmatics. In terms of the receptive-expressive distinction, the evidence suggests that receptive semantic processing (as indexed by the N400) is often atypical, while expressive phonology and syntax may be relatively preserved in some autistic individuals (Schaeffer et al. 2023). The most pronounced difficulties appear in expressive pragmatics, though receptive comprehension of non-literal language is also frequently affected. In other words, local processing seems to be intact or partially-compensatory, while global integration is altered.

Research on language in schizophrenia has historically focused on identifying so called thought disorder in adults. Studies show that patients often produce semantically correct but incoherent sentences, possibly due to disrupted fronto-temporal network integration between auditory and language areas. However, further research is required to validate and investigate this hypothesis in adolescent populations around the onset of the disorder.

Although pragmatic language traits such as frequent interruptions are core symptoms of ADHD, their neural basis remains poorly understood. Existing evidence suggests that ADHD may involve impairments in higher-order integrative processes during both receptive (e.g., speech perception in complex auditory environments) and expressive (e.g., verbal fluency) language tasks. However, a strictly critical interpretation of these findings may be premature: given the urgent need to identify reliable biomarkers for ADHD, particularly in light of ongoing concerns regarding misdiagnosis and overdiagnosis, these results warrant further investigation.

Research on adults with depressive symptoms shows that both speech acoustics and language abilities often change, such as the observed increased use of first-person pronouns and negative-connotated words. In terms of receptive language, studies have reported altered processing of emotional prosody and increased vulnerability to linguistic interference in noisy environments (Xie et al. 2019). Despite these findings, the neurobiological mechanisms behind these alterations remain unexplored, particularly in a developing population. While language abilities have sometimes been included within cognitive assessments in neuroscience studies involving people with depression, prior research rarely examined speech or language tasks during brain activity recording. As a result, the specific nature of these alterations is still unclear.

Studying the neurobiological mechanisms of those reported speech or language alterations could help better understand the cause of the change and therefore inform interventions that take language and communication abilities into account. For example, interventions might target receptive semantic integration deficits in ASD or expressive discourse coherence in schizophrenia. Most of the previous studies (often done in adults) found that there are distinct alterations between psychiatric conditions in the integration between different neural networks relevant to speech and language (e.g., integration between auditory and language networks), while systematic investigations are needed to confirm these very initial findings. This is crucial in the clinical practice, as improvements in language skills, both in form and content, are closely associated with better quality of life. When patients experience changes in their language abilities or perceive their communication as inadequate or different from that of others, these difficulties can become a source of distress.

## Limitations

This review presents some limitations. First, the available literature is rather imbalanced across disorders, with autism spectrum being substantially more represented than schizophrenia, ADHD, and depression, particularly in pediatric populations. The scarcity of studies involving children and adolescents limits conclusions regarding developmental trajectories of language processing and age-related neurobiological mechanisms.

Second, considerable methodological heterogeneity exists across studies. Investigations differ widely in participant characteristics, language and speech paradigms, task demands, and outcome measures, making direct comparisons difficult. Similarly, studies employ diverse neuroimaging techniques, including EEG, MRI, and fNIRS, each providing valuable but not directly comparable information about neural functioning. Third, the reviewed disorders themselves are highly heterogeneous, encompassing substantial variability in symptom presentation, cognitive abilities, language skills, and developmental profiles. This heterogeneity complicates efforts to identify disorder-specific neural markers of speech and language functioning.

Finally, the limited number of multimodal and longitudinal studies restricts our understanding of how language-related neural alterations emerge and evolve across development. Future research would benefit from larger samples, standardized paradigms, and longitudinal designs.

## Conclusion

New methods are rapidly developing allowing for the combination of behavioral data, speech analysis and neuroscientific methods which provide unique opportunities for better understanding associations between language and neurodevelopmental alterations in children and adolescents with a psychiatric diagnosis. Given the current paradigm shift in psychiatry focusing more on symptoms rather than syndromes, looking at language as the main transporter of feelings, psychosocial behavior and mental health is crucial. For those reasons, considering language functions in research as well as in diagnostics and behavioral interventions in psychiatry is of the utmost importance. Therefore, more systematic research on the neurobiological mechanisms underlying language changes in children and adolescents with psychiatric disorders are urgently needed.

## Data Availability

No datasets were generated or analysed during the current study.
